# Assessment of Health and Well-Being Effects Associated With the Challenging Drinking Water Situation in the Gaza Strip: Protocol for a Cross-Sectional Household Survey Study

**DOI:** 10.2196/63415

**Published:** 2024-11-29

**Authors:** Curdin Brugger, Dominik Dietler, Bassam A Abu Hamad, Tammo van Gastel, Federico Sittaro, Rodolfo Rossi, Branwen Nia Owen, Nicole Probst-Hensch, Mirko S Winkler

**Affiliations:** 1 Department of Epidemiology and Public Health Swiss Tropical and Public Health Institute Allschwil Switzerland; 2 Faculty of Medicine University of Basel Basel Switzerland; 3 Division of Occupational and Environmental Medicine Lund University Lund Sweden; 4 School of Public Health Al-Quds University Gaza Occupied Palestinian Territory; 5 International Committee of the Red Cross Geneva Switzerland

**Keywords:** drinking water, water quality, households survey, noncommunicable diseases, protracted conflict, humanitarian crisis, Gaza Strip, well-being

## Abstract

**Background:**

The water supply in the Gaza Strip, Palestine, has been unstable and under strain for decades, resulting in major issues with drinking water quality, reliability, and acceptability. In 2018, between 25% and 30% of Gazans did not have regular access to running water. The progressive deterioration of water infrastructure and concerns over the quality of piped water have resulted in a complex mix of drinking water sources used in the Gaza Strip. The challenges of safe water provision in the Gaza Strip could potentially have severe adverse effects on the population’s health and well-being.

**Objective:**

The main objectives of this survey are to determine the quality of drinking water at the household level and to investigate the association of various health outcomes with water quality at the household level in the Gaza Strip.

**Methods:**

We conducted a cross-sectional household survey in the North Gaza, Gaza, and Rafah governorates between January and March 2023. We selected a subsample of households from a representative cross-sectional survey conducted in the Gaza Strip in 2020 with persons aged 40 years and older. From each household in the 2023 survey, we invited 3 individuals (2 older than 40 years and 1 between 18 and 30 years) to participate. The face-to-face interview included questions on drinking water, mental health and well-being, self-reported diagnoses for selected diseases, use of antibiotics, and knowledge about antimicrobial resistance. Additionally, we measured each participant’s blood pressure. We sampled drinking water from each household and analyzed the samples for microbial contamination, nitrate, sodium, and mineral content.

**Results:**

In total, we visited 905 households and interviewed 2291 participants. In both age groups, more female participants were interviewed. A total of 56.60% (914/1615) were aged ≥40 years, and 58.9% (398/676) were aged between 18 and 30 years. We obtained water samples from nearly all households (902/905, 99.8%). The results are expected to be published in several papers in 2025.

**Conclusions:**

The extensive survey components, coupled with drinking water testing and building on an existing survey, allowed us to identify a broad set of potential impacts on health and well-being and to track changes over time. This study intends to identify humanitarian and development interventions that could impact the population served most. However, we completed data collection before the escalation of violence in October 2023. Given the impact of the still ongoing conflict, the initial intent of this work is no longer valid. However, the results emerging from the survey may still serve as a valuable baseline to assess the impacts of the current escalations on physical and mental health and on drinking water quality. In addition, our findings could provide important information for rebuilding the Gaza Strip in a more health-promoting way.

**International Registered Report Identifier (IRRID):**

DERR1-10.2196/63415

## Introduction

### Background

Water quality has decreased drastically in Gaza, Palestine, over the past 2 decades. While nearly all residents had access to improved water 25 years ago, between 25% and 30% of Gazans did not have regular access to running water in 2018 [1]. The destruction and deterioration of water infrastructure and concerns over the quality of piped water have resulted in a complex constellation of different types of drinking water sources being used in the Gaza Strip.

In addition to the weak infrastructure and reliability, there are major issues with drinking water quality. Only about 4% of the water from groundwater wells falls within the World Health Organization (WHO) limits for nitrate and chloride. Only 1 in 10 wells was below the threshold for nitrate (50 mg/L) and 2 in 10 wells were below the threshold for chloride (205 mg/l) [2]. Four out of 5 municipal wells did not comply with the safety threshold for salinity (250 mg/L), and most of the coastal area is affected by seawater intrusion into the aquifers [2,3]. A recent study found that groundwater salinity has increased by 31% and nitrate has decreased by 20% in some wells over the past 20 years [4]. The pollution of groundwater and drinking water with toxic chemicals, such as pesticides and heavy metals, is unknown. Approximately 70% to 80% of people in the Gaza Strip are estimated to use desalinated water as their main source for drinking and cooking [5]. However, the desalinated water that many households purchase as an alternative to groundwater is not free from contamination. Studies ranging from 2014 to 2021 found that piped water, as well as water from desalination plants, exhibited high levels of microbial contaminants, including fecal coliforms [6,7]. In a study conducted in 2018, 17% of the samples were positive for fecal coliforms [3]. A study testing water from various sources found *E coli* in 6.9% of the samples [8]. Additionally, nitrate levels in desalinated water are a concern, with 13% of samples exceeding the WHO safety threshold [9].

Climate change is likely to further exacerbate water quality and availability in the Gaza Strip with an expected increase in the average temperature between 1 and 1.5 °C by 2050 [10,11]. In the same time frame, the number of days above 30 °C is anticipated to increase by 60%, along with the increasing sea level, pushing seawater intrusion into the aquifer farther inland [10,11]. Annual rainfall could decrease by up to 20%, while extreme weather, such as heavy rainfall in winter will be more frequent, increasing the frequency of flooding events [10]. Moreover, it is expected that the southern part of the Gaza Strip will be at increased risk of chronic annual drought events due to much lower precipitation conditions than other areas in the Gaza Strip [12].

The significant microbial pollution in the Gaza Strip’s drinking water raises public health concerns. Fecal coliform poses a severe risk for the transmission of water-borne diseases, encompassing various pathogens causing mild infections like acute diarrhea to severe conditions such as chronic diarrhea, dysentery, hepatitis A, typhoid, and cholera [13]. The high microbial pollution is particularly of concern in light of the high levels of antimicrobial resistance (AMR) found in bacteria isolated from human and environmental samples taken at hospitals, farms, and coastal waters [3,14-16]. AMR further complicates the treatment of some of these infections, posing a particularly pertinent public health problem [17].

Additionally, the levels of physicochemical parameters in water sources in Gaza are a public health concern. High nitrate pollution in most drinking water sources indicates that the Gazan population is at an increased risk for a range of severe diseases, such as colorectal cancer, thyroid disease, neural tube defects, and infant methemoglobinemia [18]. Sodium levels in municipal drinking wells of the Gaza Strip are above the permissible limits for consumption [19]. An excess intake of sodium can lead to a range of adverse health effects, including obesity, hypertension and subsequent stroke, coronary heart disease, renal disease, osteoporosis, and others [20-24]. Additionally, low mineral content in drinking water, such as calcium and magnesium, has been found to have various adverse health impacts. Associated health risks include an increased risk of cardiovascular disease [25-30], caries [31,32], osteoporosis [33,34], and various cancers [35-40]. A recent study in the Gaza Strip showed an association between the consumption of desalinated water and osteoporosis [41]; this is highly relevant because of the high consumption of desalinated water in this area [5].

Beyond the physical health effects, the dire water situation in the Gaza Strip can also affect its people’s mental health and cognitive functions through different pathways [42]. Insecurity about water quality and availability, as well as potentially high water prices, can trigger stress and affect well-being. Chronic stress is linked to severe and interlinked physical and psychological health problems, including depression and anxiety disorders and associated cardiovascular diseases [43,44]. Additionally, the lack of water can cause fatigue, which impacts mood stability and cognitive function and can increase anxiety or irritability [43]. Dehydration can further contribute to difficulties in concentration, reaction time, memory, and reasoning [43,44].

### Knowledge Gaps and Objectives

The existing evidence highlights numerous potential public health risks in the Gaza Strip, with persistent knowledge gaps in associated health determinants and outcomes. Water quality is primarily assessed at the source (eg, groundwater wells and desalination plants), neglecting the mixing of water from different sources, water distribution systems, and water tanks widely used for storage. Inadequate distribution and improper storage practices can further contribute to contamination, while possible treatment at the household level before consumption can improve water quality. Evidence of the magnitude of health conditions related to low drinking water quality (eg, diarrhea, diseases related to high nitrate or sodium, and conditions related to low mineral consumption) is incomplete. Additionally, there are no data on the presence of AMR pathogens in Gazan household drinking water. There are insufficient data on practices and knowledge of antibiotics and AMR among the population, which is important to better address the multisectoral challenge of emerging AMR. Addressing these gaps in an integrated manner at the individual and household levels is crucial for targeted AMR interventions and prioritized health initiatives. Quantifying water-related health conditions will allow us to estimate the impact of the water system on public health. This improved understanding of the impact of water quality and availability on health could help in priority setting and identifying potential targeted interventions in the water sector.

Against this background, in 2019, the International Committee of the Red Cross (ICRC) initiated a health impact assessment of selected essential services (electricity, water, wastewater, and food security) in the Gaza Strip to help inform strategic priorities for its interventions. This paper presents the protocol of a household survey conducted as part of the ICRC-mandated health impact assessment in early 2023. The main objectives of the household survey are to determine what contaminants (eg, microbial contamination, nitrate, sodium, and mineral content) persist in drinking water at the household level and to investigate the health conditions (eg, kidney disease, osteoporosis, methemoglobinemia, and mental health issues) associated with water quality at the household level. Ultimately, this will provide a more comprehensive understanding of the interplay between drinking water, health conditions, and mental health in the Gaza Strip. Data collection was completed before the escalation of violence in the Gaza Strip in October 2023. While some of the findings from the survey might not be relevant anymore, the results can provide a baseline for future research. Additionally, the results and conclusions may still be relevant for other areas with similar challenges in humanitarian contexts.

## Methods

### Project Design

We conducted a cross-sectional household survey in the Gaza Strip from January to March 2023. For the 2023 survey, we revisited a subsample of households from a survey conducted in 2020. This minimized the potential burden on study participants while maximizing synergies with the existing data. Additionally, this study design allowed for more in-depth analyses, including an assessment of changes over time, as we could use selected indicators from the 2020 survey as a baseline for the 2023 survey. The 2020 survey was a representative, cross-sectional, anonymous household survey carried out by Al-Quds University, the American University in Beirut, Imperial College in London, and Juzoor, a Palestinian nongovernmental organization (NGO). It included 4576 people from 2443 households and focused on dietary patterns and noncommunicable diseases (NCDs). The study population was comprised of persons older than 40 years living in all 5 governorates of the Gaza Strip. Using a systematic cluster random sampling method, the 2017 Population and Housing Census served as the sampling frame to select enumeration areas for each governorate [45]. A total of 163 clusters proportionate to the population size of the 5 governorates were selected. In each cluster, 15 households were sampled, and in each household, 1 eligible female and 1 eligible male were interviewed [45]. The 2020 survey is described in more detail in 2 publications from Abu Hamad et al [45] and Basu et al [46].

### Ethical Considerations

Ethical approval was granted by the Helsinki Committee of the Palestinian Health Research Council in the Gaza Strip (June 6, 2022; PHRC/HC/1141/22), the Ethikkommission Nordwest- und Zentralschweiz (January 9, 2023; AO_2022-00076), and the ICRC internal Ethics Review Board (January 26, 2023; proposal no 2301_JAN and validation reference LDPCORE 23/00006-CGB/bap). The Ministry of Interior and the Ministry of Health in Gaza also reviewed and approved the survey tools and the full study protocol.

The household survey involved no collection of biological samples and only a minimal time burden on participants. The survey collected data on mental health, which can be sensitive. However, the questionnaire survey merely assessed the participants’ mental health status. No traumatic events were discussed, and no in-depth discussion of any mental health problems took place, thereby reducing the potential burden on participants. The participants received no direct benefits but were compensated with a box of dates for contributing to the survey with their time and providing a water sample.

All participants in the household survey were required to sign an informed consent form prior to participation in the study. The informed consent explained the content and purpose of the study, stated clearly that participants were free to withdraw at any time without any consequences, and informed them that the data might be used and shared with other researchers in anonymized form. Participants’ names and addresses were used only for recruitment, with access restricted to field coordinators and interviewers. Each participant was assigned an identification number, which was used for data entry, analysis, and coding of water samples. All primary data were anonymized upon entry. The anonymized data will be archived on secure servers at the Swiss Tropical and Public Health Institute for 10 years after the project’s completion.

### Study Setting, Population, and Sampling

The study was conducted in the North Gaza, Gaza, and Rafah governorates to represent the diversity of settlements in the Gaza Strip. The Gaza Governorate, with Gaza City as its capital, is the economic and administrative center of the Gaza Strip. It is home to a socioeconomically diverse population and has an extremely high population density of 13,000 inhabitants per square kilometer. In Rafah, the southernmost governorate of the Gaza Strip, a large part of the population lives in poverty, many of them refugees and rural people. Saltwater intrusion into the aquifer is particularly problematic in Rafah, and water quality parameters, such as nitrate levels, are the worst in all areas of the Gaza Strip [47]. North Gaza is frequently affected by violent escalations with Israel. Poverty levels are similar to Rafah, making the area particularly vulnerable to the health effects of poor water quality. Large parts of the land are used for agriculture, and wastewater irrigation projects are being implemented in North Gaza. The main wastewater collection facility is located in the north.

**Figure 1:**
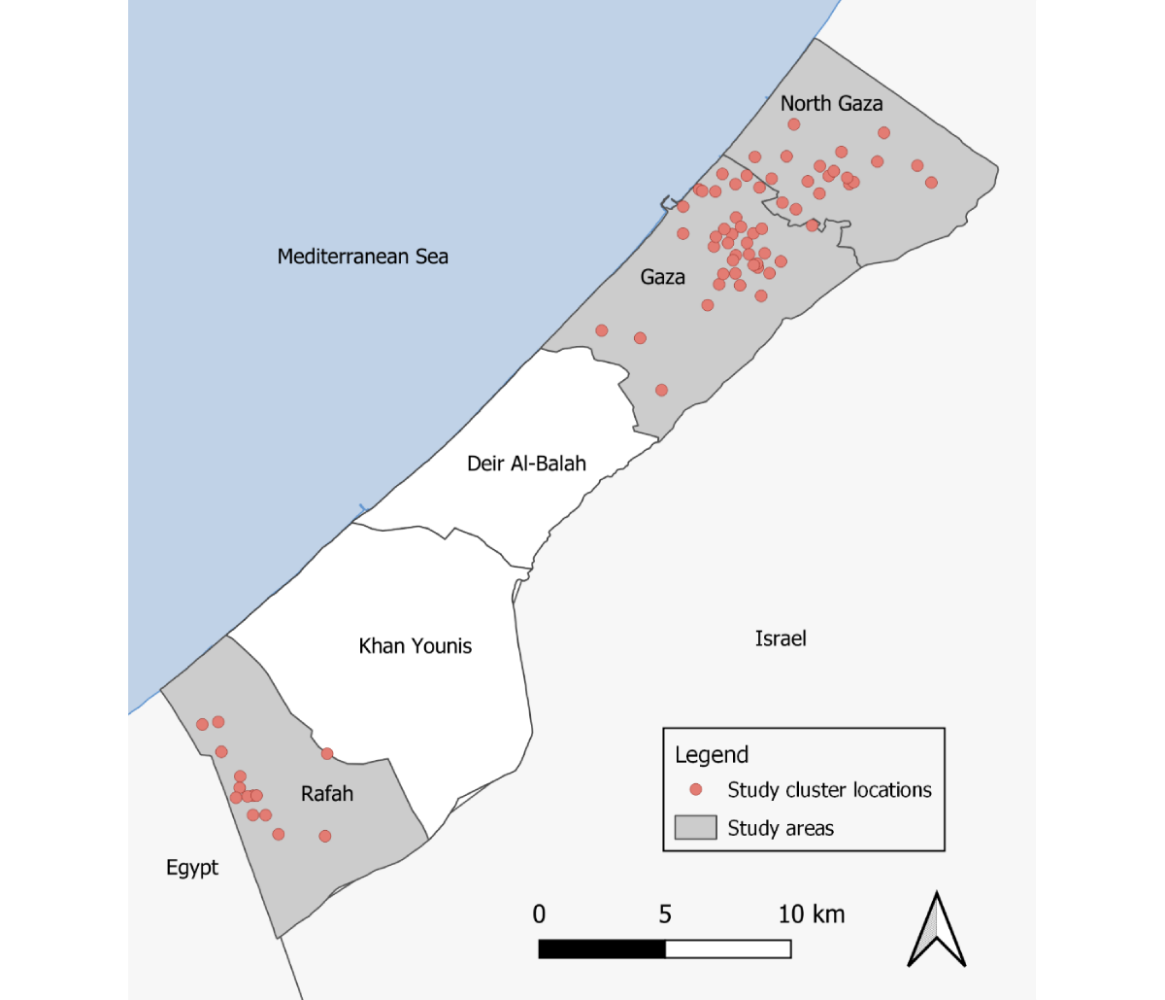
Study clusters included in the household survey in the Gaza Strip. In each cluster, up to 15 households were included in the survey.

For this survey, we sampled a subset of the previously conducted 2020 survey. We used stratified random sampling to select the study population for the 2023 survey from a complete list of households included in the 2020 survey. In total, we included 905 households proportional to the population in the 3 study areas. Figure 1 shows the distribution of clusters in the Gaza Strip visited in the household survey. For each cluster, we visited between a minimum of 12 and a maximum of 15 households and interviewed between 1 and 3 participants in each household.

We invited all respondents from the 2020 survey in the selected households to participate in the 2023 study. For a household to be eligible for inclusion in the 2023 survey, at least one of the respondents from the 2020 survey needed to be present. Otherwise, we revisited the household up to 4 times before they were considered lost to follow-up. If the original respondents had moved since the 2020 survey or refused to participate, we replaced the household with another yet unsampled household in the same cluster. If only 1 original participant was present or agreed to participate, we replaced the second respondent with a new participant over the age of 40 years in the same household.

Additionally, we randomly selected 1 respondent between 18 and 30 years from any available household members in that age group present at the time of the survey. If none were available in the initial household, an additional household in the same building was randomly selected. To select the additional household, the floor and apartment door were randomly selected. In case there was no available respondent between 18 and 30 years in the additionally selected household, the procedure was repeated. No additional respondents were selected if no respondents between 18 and 30 years of age were available in the whole building.

Before starting the study, all interviewers were trained in the interview process, the correct measurement of blood pressure, and the correct collection of water samples. Additionally, the questionnaire was piloted to ensure participants understood the questions correctly. Two field workers visited each household. All field workers were female and had been part of the 2020 survey team. Having female interviewers visiting the households made participation for female household members easier and more comfortable, as they could remain without a headscarf. The survey was administered face-to-face, with the interviewer capturing data electronically on a tablet.

The sample size calculation was based on the objective of estimating the prevalence of various NCDs. For the questionnaire survey, we assumed a prevalence of 50% as the most conservative assumption. The respondent groups (ie, females and males above 40 years and additional household members between 18 and 30 years) will be analyzed separately. Therefore, the resulting sample size indicates the required number of individuals in each group. The sample size was calculated using the following formula:

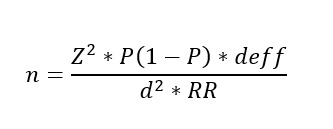



where Z is the z-value (1.96, corresponding to a 95% CI), P is the expected prevalence (50%), d is the precision (5%), deff is the design effect (2), and RR is the response rate (0.9), based on nonresponse rate in the baseline survey (96.6%) and the estimated proportion of respondents remaining at the same location since the baseline survey (95%). The resulting sample size was 854 households, which we rounded up to 900, corresponding to 2700 respondents (1800 aged 40 years and older and 900 aged 18 to 30 years), in the best-case scenario.

### Survey Modules

**Figure 2:**
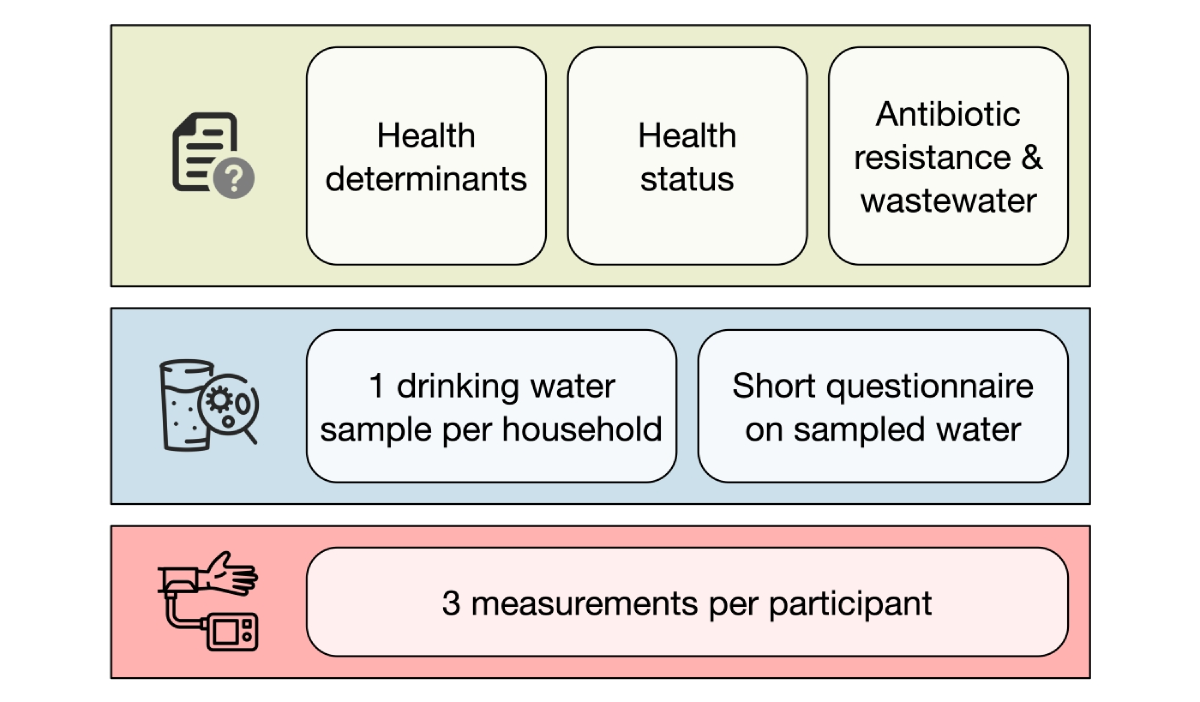
The 3 modules of the 2023 household survey and their components: questionnaire (green), drinking water sample (blue), and blood pressure measurements (red).

The 2023 household survey consisted of three modules: (1) a questionnaire, (2) blood pressure measurements, and (3) a drinking water sample. Figure 2 displays an overview of the 3 modules and the components of each.

The questionnaire consisted of 3 major components, which are outlined in Textbox 1.

When applicable, we used and sometimes adapted established questionnaires and survey items to build this questionnaire. Table 1 lists the different questionnaires used for the household survey. For most of the questionnaire, not all the questions were applied in our household survey. The column *survey items included in our questionnaire* lists the items from the respective questionnaire that we adopted and included in the survey.

**Components of the questionnaire.**

1. Health determinants: drinking water source, storage, and consumption; recreational activities (eg, swimming in the sea) and perception of the environment; and social network
2. Health status: self-reported mental health and well-being, and self-reported diagnoses/diseases for selected noncommunicable diseases (NCDs) and communicable diseases, including reports of diarrheal symptoms among participants and children under 5 years in the household, as reported by parents
3. Consumption of antibiotics, knowledge about antibiotics and antimicrobial resistance (AMR), attitudes toward wastewater irrigated crops, and perception of the environment on the influence of health

**Table 1:** Overview of established questionnaires used in the household survey questionnaire.

Questionnaire name	Survey items included in our questionnaire	Source
Multiple Indicator Cluster Surveys (MICS): Water quality testing questionnaire	Water sources for Drinking Cooking Hygiene and bathing Treatment of water at the household level	UNICEF^a^, MICS6^b^ Questionnaire, Water Quality Testing Questionnaire, 2020 [48]
Household Water Insecurity Access Scale (HWIAS)	Water availability and quantity Water safety and quality Emotions related to water access and security	Tsai et al (2016) [49]
The WHO^c^ STEPwise approach to noncommunicable disease risk factor surveillance	History of selected NCDs^d^: Raised blood pressure Diabetes Raised total cholesterol Cardiovascular diseases Chronic respiratory diseases Cancer	Household survey 2020: Abu Hamad et al (2022) [45]; Sibai et al (2009) [50]; WHO STEPs [51]
WHO Oral Health Questionnaire for Adults	Oral health and hygiene Dental health and hygiene	WHO, Oral Health Surveys - Basic Method (2013) [52]
Questionnaire on Self-Reported Skin Complaints	Skin complaints and irritations Skin diseases	Dalgard et al (2003) [53]
General Health Questionnaire 12-Items (GHQ-12)	Mental disorders Mental health problems Mental disturbances Psychosocial health	Anjara et al (2020), Goldberg et al (1997), Goldberg and Hillier (1979) [54-56]
Health-Related Quality Of Life (WHOQOL-BREF)	Quality of life and well-being Individuals’ perceptions of their position in life	WHO Program on Mental Health-WHOQOL User Manual, 1998 [57]
Antibiotic Resistance: Multicountry Public Awareness Survey	Antibiotics use and knowledge Antimicrobial resistance knowledge	WHO, Antibiotic resistance: multicountry public awareness survey (2015) [58]
Attitudes Toward Wastewater Reuse	Attitude toward wastewater reuse and wastewater irrigated crops Perception of water and environment	Friedler et al (2006) [59]

^a^UNICEF: United Nations Children's Fund.

^b^MICS6: Multiple Indicator Cluster Survey, 6th round.

^c^WHO: World Health Organization.

^d^NCD: noncommunicable disease.

Following the interview, we measured each participant’s blood pressure using oscillatory blood pressure monitoring devices. For each participant, we took 3 measurements with a break of at least 3 minutes between each measurement. We asked participants to follow a standardized protocol to ensure standardized blood pressure measurements. Participants had to refrain from eating or drinking for at least 30 minutes and ensure their bladder was empty before the measurement. Prior to the measurement, participants had to sit comfortably in a supported position for a minimum of 5 minutes. During the measurement, they had to keep both feet flat on the ground, with their legs uncrossed. The arm with the cuff was rested at chest height on a table and the cuff was put on bare skin. Finally, there was no talking during the measurement to minimize potential disturbances. The field workers advised participants with high blood pressure (systolic blood pressure above 140 mmHg or diastolic blood pressure above 90 mmHg) to visit a doctor for further assessment.

Additionally, we collected a drinking water sample in all households for subsequent analyses. The samples were tested for 9 common water quality parameters (Table 2) by the Coastal Municipalities Water Utility laboratory in Gaza City. We took the sample from the source the respondents usually use for drinking. The field workers collected approximately 100 ml using sterile containers and stored the samples in a cool box between 4 and 6 °C. Within 6 hours, the samples were transported to the laboratory and analyzed within 30 hours of collection. We collected an additional sample from 10% (90/902) of the households and analyzed the sample at a different laboratory for quality control. In each household, we administered a brief questionnaire to collect data regarding the water source and any filtration or purification methods used in the household. The questions were based on the water quality testing questionnaire from the Multiple Indicator Cluster Survey, 6th round (MICS6) [48].

**Table 2:** Overview of measured water quality parameters.

Water quality parameter	Abbreviation	Unit
Total coliform	TC	CFU/100 ml
Fecal coliform	FC	CFU/100 ml
Nitrate	NO_3_-N	mg/L
Sodium	Na^+^	mg/L
Calcium	Ca^2+^	mg/L
Magnesium	Mg^2+^	mg/L
pH	pH	N/A^a^
Electrical conductivity	EC	µS/cm
Total dissolved solids	TDS	mg/L

^a^N/A: not applicable.

Among households whose water tested positive for fecal coliform in the first round of sampling, we selected 90 for a second round of water sampling. The selection was based on high fecal coliform counts, geographic distribution, and drinking water sources. We took the second sample 4 weeks after the first sample and analyzed it for AMR bacteria. Of these 90 households, we selected 10 (11%) for additional water chain testing. Starting from the point where household members accessed their drinking water, we followed the water chain and took a sample at each potential contamination site. Examples of sample locations are the kitchen faucet, the rooftop storage tank, the pipe from a water tanker truck, and the refill pipe at a desalination plant. The number and location of water samples taken varied for the water chain testing depending on the drinking water source used in the households.

### Data Analysis

Data analysis will be performed with R and RStudio software (R Foundation for Statistical Computing) [60,61]. We will include questionnaires with missing data as long as 75% of the questionnaire has been filled out. When using data from the 2020 and 2023 surveys, we will only include participants who took part in both surveys. As the first step, we plan to focus mainly on descriptive analyses to give an overview of the data gathered. We will treat water quality variables as continuous variables and compare them to national and international water quality guidelines. If needed, we will apply log transformations to meet normality distribution requirements. Further, we will compare the results of the water analysis across different water sources and investigate potential reasons for observed differences between water sources. In the second analysis step, we will compare water quality and water-related health conditions. This will include integrating data from the 2020 survey to investigate changes in health status over time. We will analyze health determinants (other than water quality) and health outcomes as either dichotomous (eg, presence of diabetes) or categorical (eg, type of water treatment) using Pearson chi-square tests to compare groups. We will use linear or logistic regression models to explore associations between health determinants and outcomes, depending on whether the outcome variable is continuous or dichotomous. We will specifically assess associations such as microbial pollution with diarrheal disease, nitrate levels with cancer and kidney disease, and low mineral levels with osteoporosis. Since our analyses are hypothesis-driven, we will not apply adjustments for multiple testing. Additionally, we intend to delve into the mental health burden among residents in both the 2020 and 2023 surveys, aiming to investigate temporal trends or disparities. We will explore the determinants of mental health within the Gaza Strip, considering various socioeconomic, environmental, and psychosocial factors that could influence mental well-being by including data from the 2020 and 2023 survey and external data sources such as data from ACLED (Armed Conflict Location and Event Data) [62]. As part of the health impact assessment, we collected additional data on AMR in different sectors of the Gaza Strip. We will combine these findings with the AMR data from household drinking water and survey responses on antibiotic use and AMR awareness to provide a clearer overview of the scale of the AMR issue in the Gaza Strip. Taken together, these analyses will provide a comprehensive understanding of the complex interplay between water quality, health outcomes, and mental well-being in the Gaza Strip.

## Results

**Figure 3:**
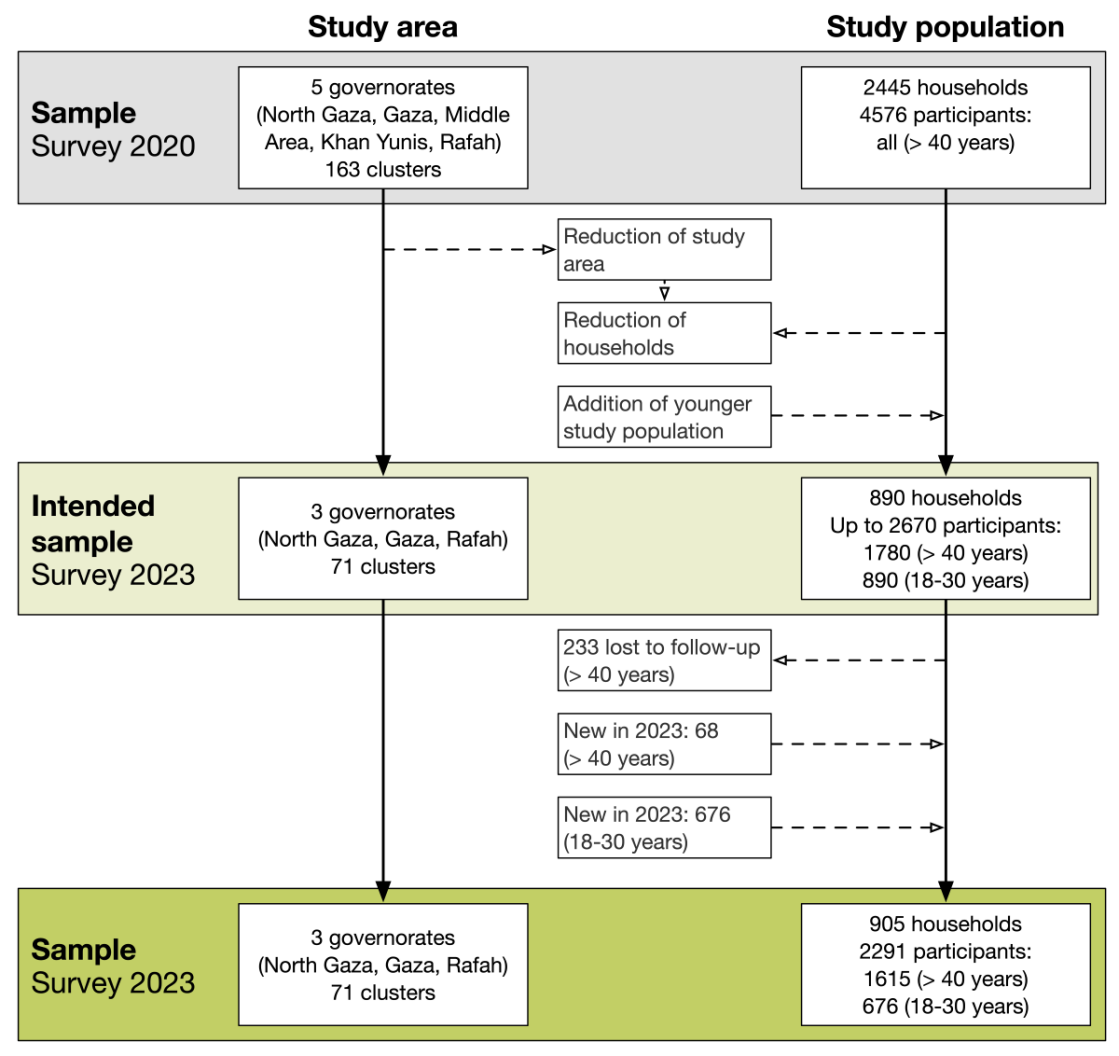
Study area and population in the 2020 and 2023 survey.

The household survey was initiated on January 24, 2023, and the final interviews were administered on March 7, 2023. We visited a total of 905 households, interviewed 2291 participants, and collected water samples in 99.7% (902/905) of the households (Figure 3). Data analysis was still ongoing at the time of submission. We plan to conclude the data analysis in early 2025 and expect to publish the results in several peer-reviewed papers in the course of 2025.

Table 3 shows an overview of the participants’ characteristics from the 2023 household survey compared with those from the 2020 survey. In 2023, 70.50% (1615/2291) of the participants were aged 40 years and older, and 29.51% (676/2291) were between 18 and 30 years old. Moreover, 95.79% (1547/1615) of the participants aged 40 years and older in 2023 were already part of the 2020 survey. The follow-up rate in 2023 was 77.01% (1615/2097) compared to the 2020 sample in the same study area. In both age groups in 2023, more female participants were interviewed, with 914 out of 1615 (56.6%) in the first group and 398 out of 676 (58.9%) in the second group. This is similar to the participants of the 2020 survey, where 53.13% (1071/2016) were female. A more detailed overview of participant characteristics by governorate is available in Table S1 in Multimedia Appendix 1.

**Table 3:** Description of participants in the 2023 household survey and comparison with participants in the 2020 survey.

Characteristic		Study year		
		2023		2020
Number of households		905		1065
Number of participants		2291		2016
≥**40 years age group**				
	Number of participants	1615	2016	
	Same as 2020, n (%)	1547 (95.79)	—^a^	
	Female, n (%)	914 (56.60)	1071 (53.13)	
	Age, mean (years), min-max	59 (40-89)	57 (40-118)	
	Refugee, n (%)	1044 (64.64)	1332 (66.07)	
**18-30 years age group**				
	Number of participants	676	—	
	Female, n (%)	398 (58.9)	—	
	Age, mean (years), min-max	23 (18-30)	—	
	Refugee, n (%)	419 (62)	—	

^a^Not applicable.

## Discussion

### Expected Findings

This paper describes the design and methodology of a household survey conducted in the Gaza Strip between February and March 2023. We conducted a survey in 905 households with 2291 participants and collected 902 drinking water samples in the Gaza Strip, achieving a follow-up rate of 77.01% (1615/2097) with the participants from the 2020 survey.

Using the results from the water samples, complemented with recent data on groundwater quality, we aim to provide a comprehensive overview of the water quality in the Gaza Strip in early 2023 and identify potential risks for public health. Previous studies and measurements suggest high levels of chemical and some microbial contamination in groundwater wells and the drinking water system [3,6,7]. We expect to find similar results in our measurements at the household level, but in contrast to previous studies, we can directly link water quality to health at the individual level. The widespread use of desalinated water may result in low levels of essential minerals such as magnesium and calcium, which may have implications for public health. However, this issue has only been investigated in a few studies on the Gaza Strip. Compared to the 2020 survey, we expect only an increase in the prevalence of NCDs that corresponds to the 3-year increase in the study population’s age. Having data on the mental health status of our study populations from 2020 and 2023 will allow us to study the mental health change over this time. Findings from previous studies report a high level of mental health burden in the population, and we assume our results to be similar for mental health [63,64]. Taken together, the upcoming results of our project will provide a thorough understanding of the relationships between water quality, health, and mental well-being in the Gaza Strip, as they were before the current escalation of violence.

### Strengths and Limitations

One strength of our study lies in integrating data from a previous survey on the participants’ health status. This approach allows us to observe trends over time and gain a more detailed understanding of the health burden within the Gaza Strip. This will help us identify a broad set of potential impacts on health and well-being and track changes over time. An additional strength is our sampling of water at the household level, which allows us to test the water that the participants consumed rather than sampling further up the water chain, after which contamination could occur before consumption.

Our study also has several limitations. The main limitation is the cross-sectional design of the survey. While we have data from 2020 and 2023, we only have data from one survey for several important questions. Most of the health outcomes included in the survey are NCDs, which develop over the course of a person’s life. We lack data on long-term water quality from several years back, as well as data on health outcomes prior to 2020. This temporal alignment means that we cannot establish causation between water quality and health outcomes based on the current dataset. All health information collected in the survey is self-reported and not from medical diagnoses. To limit wrong answers, we used validated questionnaires that have been used in many contexts to screen for NCDs and other health statuses.

The survey was completed in March 2023 before the escalation of violence in October 2023. Many houses and much of the infrastructure have been destroyed, negatively impacting the physical and psychological health of the residents of the Gaza Strip. While the situation in the Gaza Strip has completely changed and some of the findings may not be relevant anymore, our study could serve as a valuable baseline for future research evaluating the impacts of the escalations. Furthermore, the findings from this survey may still be relevant for other areas with similar challenges linked to drinking water in humanitarian contexts.

### Conclusions

The data collected in this survey will provide valuable insights into drinking water quality at the household level and its potential impact on the health and well-being of people in the Gaza Strip as the situation stood in early 2023. The results emerging from this study may serve as a baseline for future research that seeks to assess the impact of the current escalations on water quality and physical and mental health. Additionally, the results from this study can provide information regarding the water system from a public health perspective, which could be useful in rebuilding the Gaza Strip. Collaborative efforts involving government agencies, NGOs, and international partners will be essential in addressing the complex challenges facing water, sanitation, and public health in this process.

## Appendix

Multimedia Appendix 1 Participant characteristics in the 2020 and 2023 surveys by governorate.

## Abbreviations

ACLED: Armed Conflict Location and Event Data
AMR: antimicrobial resistance
ICRC: International Committee of the Red Cross
MICS6: Multiple Indicator Cluster Survey, 6th round
NCD: noncommunicable disease
NGO: nongovernmental organization
UNICEF: United Nations Children’s Fund
WHO: World Health Organization

## Data Availability

The data collected and generated during data collection and analysis of this study will be available from the corresponding author upon reasonable request.
