# Supplementary material for: Assessment of Health and Well-Being Effects Associated With the Challenging Drinking Water Situation in the Gaza Strip: Protocol for a Cross-Sectional Household Survey Study
Source: JMIR Res Protoc. 2024 Nov 29;13:e63415. doi: 10.2196/63415 (PMC11645501; doi:10.2196/63415)
Supplement: Multimedia Appendix 1 [file resprot_v13i1e63415_app1.docx]

## Appendix

*Table A1: Participant characteristics in the 2023 and 2020 survey by governorate.*

| **Study year** | **2023** | | | | **2020** | | | |
| --- | --- | --- | --- | --- | --- | --- | --- | --- |
| **Study area** | **All** | **North Gaza** | **Gaza** | **Rafah** | **All** | **North Gaza** | **Gaza** | **Rafah** |
| **Number of households** | 905 | 254 (28%) | 469 (52%) | 182 (20%) | 1’065 | 285 (27%) | 570 (54%) | 210 (20%) |
| **Number of participants** | 2’291 | 669 (29%) | 1’141 (50%) | 481 (21%) | 2’016 | 516 (26%) | 1‘093 (54%) | 407 (20%) |
| **Age group** | **40+** | | | | **40+** | | | |
| **Number of participants** | 1’615 | 464 (29%) | 817 (51%) | 334 (21%) | 2’016 | 516 (26%) | 1‘093 (54%) | 407 (20%) |
| **Same as 2020** | 1’547 (96%) | 437 (94%) | 785 (96%) | 325 (97%) | – | – | – | – |
| **Female** | 914 (57%) | 251 (54%) | 477 (58%) | 186 (56%) | 1’071 (53%) | 276 (54%) | 582 (53%) | 213 (52%) |
| **Age** [Mean(min-Max)] | 59 (40 – 89) | 58 (40 – 88) | 60 (40 – 89) | 58 (40 – 89) | 57 (40 – 118) | 56 (40 – 87) | 58 (40 – 90) | 57 (40 – 118) |
| **Refugee** | 1044 (65%) | 363 (78%) | 420 (51%) | 261 (78%) | 1332 (66%) | 408 (79%) | 603 (55%) | 321 (79%) |
| **Age group** | **18-30** | | | | – | | | |
| **Number of participants** | 676 | 205 (30%) | 324 (48%) | 147 (22%) | – | – | – | – |
| **Female** | 398 (59%) | 129 (63%) | 194 (60%) | 75 (51%) | – | – | – | – |
| **Age** [Mean(min-Max)] | 23 (18 – 30) | 23 (18 – 30) | 23 (18 – 30) | 23 (18 – 30) | – | – | – | – |
| **Refugee** | 419 (62%) | 146 (71%) | 163 (50%) | 110 (75%) |  |  |  |  |
